# Urbanization causes biotic homogenization of woodland bird communities at multiple spatial scales

**DOI:** 10.1111/gcb.16350

**Published:** 2022-08-14

**Authors:** William Sidemo‐Holm, Johan Ekroos, Santiago Reina García, Bo Söderström, Marcus Hedblom

**Affiliations:** ^1^ AgriFood Economics Centre Swedish University of Agricultural Sciences Lund Sweden; ^2^ Centre for Environmental and Climate Science Lund University Lund Sweden; ^3^ Department of Agricultural Sciences University of Helsinki Helsinki Finland; ^4^ Royal Swedish Academy of Sciences Stockholm Sweden; ^5^ Department of Urban and Rural Development Swedish University of Agricultural Sciences Uppsala Sweden

**Keywords:** beta diversity, biodiversity, birds, city, forest, red‐list, urbanization, woodland

## Abstract

Urbanization is a major contributor to biodiversity declines. However, studies assessing effects of urban landscapes per se (i.e., disentangled from focal habitat effects) on biodiversity across spatial scales are lacking. Understanding such scale‐dependent effects is fundamental to preserve habitats along an urbanization gradient in a way that maximizes overall biodiversity. We investigated the impact of landscape urbanization on communities of woodland‐breeding bird species in individual (local scale) and across multiple (regional scale) cities, while controlling for the quality of sampled habitats (woodlands). We conducted bird point counts and habitat quality mapping of trees, dead wood, and shrubs in 459 woodlands along an urban to rural urbanization gradient in 32 cities in Sweden. Responses to urbanization were measured as local and regional total diversity (γ), average site diversity (α), and diversity between sites (β). We also assessed effects on individual species and to what extent dissimilarities in species composition along the urbanization gradient were driven by species nestedness or turnover. We found that landscape urbanization had a negative impact on γ‐, α‐, and β‐diversity irrespective of spatial scale, both regarding all woodland‐breeding species and red‐listed species. At the regional scale, dissimilarities in species composition between urbanization levels were due to nestedness, that is, species were lost with increased landscape urbanization without being replaced. In contrast, dissimilarities at the local scale were mostly due to species turnover. Because there was no difference in habitat quality among woodlands across the urbanization gradient, we conclude that landscape urbanization as such systematically causes poorer and more homogeneous bird communities in adjacent natural habitats. However, the high local turnover and the fact that several species benefited from urbanization demonstrates that natural habitats along the entire urbanization gradient are needed to maintain maximally diverse local bird communities.

## Graphical abstract

We surveyed woodland birds along an urbanization gradient of 32 cities in Sweden to investigate the effect of landscape urbanization on local and regional diversity. Overall, urbanization systematically caused poorer and more homogeneous bird communities in natural woodland habitats, irrespective of spatial scale. Because of a systematic loss of species, woodlands in urban areas were less important than rural woodlands to the regional diversity. However, because of a high species turnover along the urbanization gradient at the local scale, and several species being benefited by urbanization, urban woodlands proved to be essential for the local species diversity.

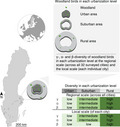

## 1 INTRODUCTION

Urban expansion into natural habitats is a significant driver of local and global biodiversity loss and biotic homogenization (Elmqvist et al., 2013; McKinney, 2006). With the total urban area projected to more than double by 2050 (Zhou et al., 2019), understanding the impact of urbanization on species communities is increasingly important for biodiversity conservation.

Birds are one of the most studied taxonomic groups regarding how urbanization affects diversity and community composition (e.g., Chace & Walsh, 2006; Marcacci et al., 2021; Melles et al., 2003; Taylor et al., 2016). Urbanization can affect bird species differently, for example, depending on functional traits such as diet (Callaghan et al., 2020; Marcacci et al., 2021) and nesting site preference (Hedblom & Söderström, 2010), yet most species respond negatively to urbanization (Carvajal‐Castro et al., 2019; Dunford & Freemark, 2005; Melles et al., 2003; Schneiberg et al., 2020). This is due to several concomitant processes, including noise and light pollution, habitat fragmentation, and the replacement of natural vegetation and resources with impervious surfaces, lawns, and popular cultivated plant species (Gilbert, 2012; Groffman et al., 2014).

To preserve bird diversity in habitats within and around cities, it is important to understand how urbanization affects the total species richness (γ‐diversity) via changes in species richness per site (α‐diversity) and the difference in species composition between sites (β‐diversity; Socolar et al., 2016). α‐diversity is typically affected by drivers operating at the scale of individual sites, such as the habitat and landscape quality. β‐diversity is instead affected by how drivers vary among sites, for example, the overall environmental heterogeneity (Socolar et al., 2016).

The impact of urbanization can depend on the spatial scale α‐ and β‐diversity are measured at. For instance, a given city generally provides an array of habitat types, for example, gardens, wetlands, and woodlands, that can support a variation of species with different habitat preferences (Aronson et al., 2017). However, environmental conditions, such as vegetation, topography, and climate, are often more similar across cities than rural areas (Dronova, 2017; Groffman et al., 2014), leading to comparatively similar environments and thus species composition across cities (Ferenc et al., 2014). Thus, because effects may differ, it is important to study the impact of urbanization on α‐ and β‐diversity at both the local (per city) and regional (across cities) spatial scale to facilitate conservation across spatial scales (Boyd et al., 2008). Although there is an increasing number of studies on birds and urbanization, few have investigated the effects of urbanization on γ‐, α‐, and β‐diversity at different spatial scales. Moreover, most studies at the regional scale have compared different habitats (e.g., urban parks, gardens, and forest remnants) and do not disentangle the effects of landscape urbanization from those of the focal habitat (e.g., Aronson et al., 2014; Ferenc et al., 2014).

To better understand the causal mechanisms that drive differences in diversity across an urbanization gradient, differences in occurring species (dissimilarity) can be partitioned into species nestedness and species turnover. Nestedness reveals a systematic loss of species (Wright & Reeves, 1992), for example because habitat specialists or poorly dispersing species disappear with increasing urbanization (Gaston & Blackburn, 2008). Turnover instead shows that dissimilarity in species composition is caused by distinct species inhabiting habitats in different levels of urbanization (see Baselga, 2012), indicating that habitats in different urbanization levels contribute to the total species pool.

An understanding of how landscape urbanization affects different components of diversity and dissimilarity at multiple spatial scales can help when designing conservation strategies, such as prioritization when protecting natural habitats from urban expansion and densification. Yet, there is a lack of studies addressing these aspects, and especially doing so simultaneously to better understand the biological processes at different spatial scales that ultimately affect the total species richness and composition.

In this study, we compared how bird diversity of woodland‐breeding species, as well as red‐listed species, measured at different spatial scales, change along a gradient of urbanization that ranges from central urban woodlands to woodlands 5 km outside the city limit. We controlled for local habitat quality of the woodlands and could thus assess the impact of landscape urbanization independently of habitat quality. Furthermore, we explored if differences in species composition along the urbanization gradient were due to species nestedness (communities in more urbanized contexts are a subset of communities in less urbanized contexts) or species turnover (communities consist of different species because species in one of the compared urbanization levels are replaced by other species). Finally, we analyzed which individual bird species were most affected by urbanization. We used a well‐replicated dataset on birds (Hedblom & Söderström, 2010), collected in 459 forest remnants (woodlands) located in urban, suburban and rural areas of 32 cities in Sweden. To our knowledge, no other study has shown the impact of landscape urbanization per se on bird communities using a stratified sampling design allowing comparisons across multiple spatial scales and cities. Our study thus provides novel insights on the role of spatial scale for how landscape urbanization affects bird diversity and species composition.

## 2 MATERIALS AND METHODS

### 2.1 Study area

**FIGURE 1:**
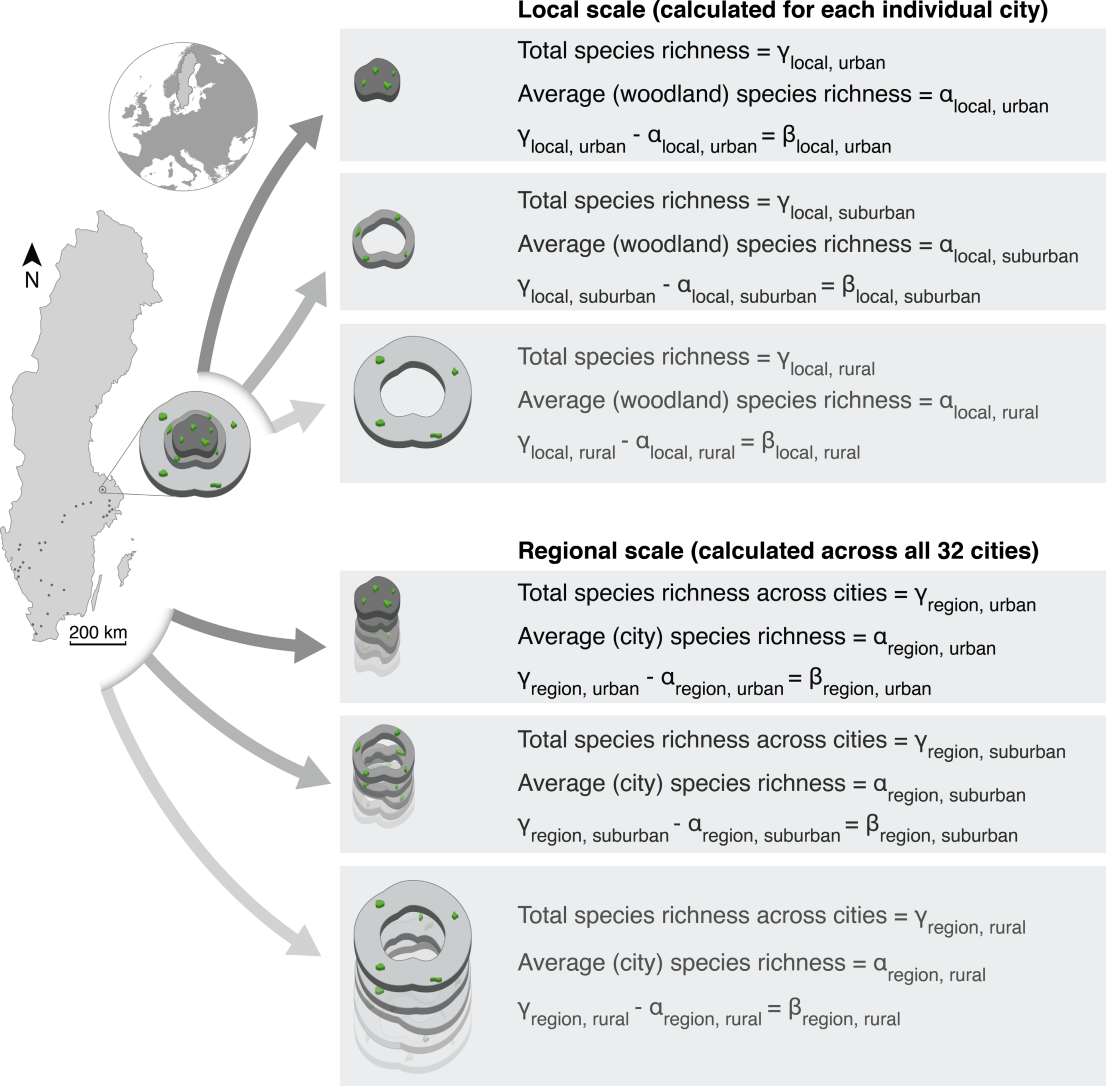
Map showing Sweden and the location of the 32 cities (dark dots) where surveys were conducted, and a conceptual illustration of how we estimated γ‐, α‐, and β‐diversity for each of the urbanization levels at the local and regional scale. The conceptual illustration shows woodlands (green) located in the urban (darkgrey shape), suburban (middle‐grey ring), and rural areas (light‐grey ring).

The study was carried out in and around 32 of the largest cities (10,000–1,150,000 inhabitants; Table S1) in southern Sweden (Figure 1). All cities were located south of 61°N in the nemoral and hemi‐boreal vegetation zones (Ahti et al., 1968). Cities further north were excluded to reduce variation in bird species composition driven by, for example, climate and vegetation zones. The study area covered 105,000 km^2^, was inhabited by 7.6 million people and characterized by forest and agricultural land covers in addition to the cities (SCB, 2006). We measured the proportion of land covered by woodlands within and around (0–5 km from the city limit) the studied cities using ArcView GIS 3.3 and the Green map of Sweden, a land‐cover map with a position tolerance of 10 m produced by digitizing objects that are identified in orthophotos (National Land Survey of Sweden, 2001).

### 2.2 Urbanization gradient

We surveyed birds in 459 individual woodlands along an urban to rural gradient in the 32 cities (see below for details). We defined the urbanization gradient based on three levels of urbanization intensity: urban, suburban, and rural area (Figure 1). Woodland sites in the urban and suburban areas were situated within the city limit, whereas rural sites were situated outside the city limit. The urban and suburban areas of all cities were characterized by built‐up area of continuous settlement, where the distance between houses was <200 m (Dijkstra & Poelman, 2014; Ode & Fry, 2006; SCB, 2001). We defined urban areas as the inner 75% of the cities' area and suburban areas as the outer 25% of the cities' area (Figure 1). We distinguished between urban and suburban areas based on that cities in Sweden usually have a share of approximately 25% characterized by residential areas with lower buildings, which are typically located close to the city limit. Consequently, suburban areas usually have larger shares of green spaces (e.g., trees, bushes and lawns) and less impervious surfaces as opposed to industrial and commercial areas, which are typically located in the city center. We selected woodlands in the rural area located within 5 km from the city limit (see Hedblom & Söderström, 2008). We set 5 km as the outer limit for the rural areas to ensure that we would find enough woodlands and that rural areas around surveyed cities did not overlap with urban or suburban areas of any neighboring city.

### 2.3 Sampled woodlands

We defined woodlands as forest stands structurally similar to natural forests, where the field layer was not managed as in parks (Lehvävirta & Rita, 2002). In Sweden, urban woodlands commonly consist of remains from non‐urban forests or forested pastures in a late succession stage, with a high proportion of old trees (Rydberg & Falck, 2000).

We used ArcView GIS 3.3 and the Green map of Sweden to identify and select woodlands larger than 1 ha along the urbanization gradient. To facilitate comparison between urbanization levels, we selected the same number of woodlands in the urban, suburban and rural area of each city. To control for the influence of habitat area and quality on bird diversity, we selected woodlands of similar size and composition (coniferous or mixed deciduous and coniferous) along the urbanization gradient of each city. This resulted in a final sample size of 459 woodland sites, with three to six woodlands in each urbanization level depending on the city.

To further assess the habitat quality along the urbanization gradient, we visited each woodland and measured a set of woodland features that commonly affect bird diversity (Nielsen et al., 2014), including Shannon's diversity index for trees (based on trees with a trunk diameter larger than 15 cm at 1.5 m above the ground [DHB]), amount of dead wood (based on the number of dead trees with a DHB larger than 15 cm), and number of shrubs (based on vegetation with a maximum stem diameter of 4 cm and height between 50 cm and 3 m). We sampled the woodland features in a circular plot with the radius of 10 m in the center of each woodland, as per the International Forestry Resources and Institutions Field Manual (Wertime et al., 2008). This allowed us to retrieve a representative sample of woodland interior habitat features, while minimizing confounding edge effects. We ensured that all plots had a continuous canopy and an unmanaged field layer by allowing the plot to be placed up to 50 m from the center, for example, in case there was a glade or an artificial structure in the center.

### 2.4 Bird survey

We designed a rigorous sampling method to reduce variability in bird detectability (Nichols et al., 2009). This included only sampling sites with the same habitat (i.e., continuous forest cover). We used repeated point counts by surveying all woodlands for birds three times, once in each of the periods 5–20 April, 5–20 May and 5–20 June in year 2004. We changed the order of sampled woodlands among urban, suburban, and rural areas between the survey rounds. We delayed the survey period in the eastern cities (also located at more northerly latitudes) to compensate for that long‐distance migrating species arrive later in the season. All surveys were done between sunrise and 3–5 h later, which is the period of the day with the highest bird activity. We sampled all selected woodlands of a city (those in the urban, suburban, and rural area) in the same morning. We did not survey birds when the wind speed exceeded 5 ms^−1^ nor when it was raining heavily. After arriving to a survey site, we waited for 2 min before starting the point count to let the birds calm down. All surveys lasted for 10 min during which we counted seen or heard birds, except those flying high above the tree canopy, within a radius of 50 m to have a high probability of detection without high uncertainty. Later we excluded observations of species that do not breed in forest/woodland habitats (BirdLife International, 2022). By adopting this sampling method, we ensured that variation in detection probabilities was maintained low (see also Hedblom & Söderström, 2010). Data from the surveys are stored at the data repository Dryad and can be accessed at (Hedblom et al., 2022).

### 2.5 Estimation of diversity and dissimilarity components

Before any analysis, we aggregated the bird data across the three survey rounds per woodland to obtain a presence‐absence list of species for each of the 459 surveyed woodlands. To assess how bird diversity at different spatial scales related to landscape urbanization level, we used an additive diversity partitioning approach, where total diversity at a set scale (γ) is partitioned into the average site diversity (α) and the diversity attributable to differences in species composition between sites (β; Whittaker, 1960). Thus, we used the additive β‐diversity defined as β = γ−α (Lande, 1996), which shows the absolute difference in species among communities. Higher β‐diversity indicates larger heterogeneity in species composition, and vice versa.

The γ‐, α‐, and β‐diversity of urbanization levels were calculated at the regional and local scale as demonstrated in Figure 1 and explained below. For the regional scale, we calculated the total species richness for each urbanization level across the 32 cities (γ_region,urban_; γ_region,suburban_; γ_region,rural_). The total species richness of each urbanization level was then partitioned into the average species richness per city (α_region,urban_; α_region,suburban_; α_region,rural_) and the absolute species difference among cities (β_region,urban_; β_region,suburban_; β_region,rural_). In addition to calculating the diversity estimates for all woodland‐breeding species, we used the same approach for a subset with red‐listed species (i.e., threatened or near threatened species) based on the Swedish Red list 2005, which follows the IUCN Red list Criteria (Gärdenfors, 2005). We used the red list from 2005 as it described the status of the species at the time of the surveys.

For the local scale, we calculated total species richness for each urbanization level separately for each of the 32 cities (γ_local,urban_; γ_local,suburban_; γ_local,rural_). For each city, the total species richness in each urbanization level was then partitioned into the average species richness per woodland in the different urbanization levels (α_local,urban_; α_local,suburban_; α_local,rural_) and the absolute species difference among woodlands (β_local,urban_; β_local,suburban_; β_local,rural_). This was done with all woodland‐breeding species and the subset with only red‐listed species.

To gain further insight into how urbanization affects species composition at different spatial scales, we calculated the pairwise Jaccard dissimilarity and its components nestedness and turnover. The pairwise Jaccard dissimilarity expresses the proportion of species that is only found in one of two compared communities (Jaccard, 1912). Thus, at the regional scale, Jaccard dissimilarity (Dissimilarity_region_) was calculated between two urbanization levels at a time across all 32 cities. To compare to what degree dissimilarity was driven by nestedness versus turnover, we partitioned each estimate of pairwise Dissimilarity_region_ into its components Nestedness_region_ and Turnover_region_. Similar to the regional scale, the pairwise Jaccard dissimilarity was at the local scale (Dissimilarity_local_) calculated between two urbanization levels at a time, but separately for each city. The pairwise Dissimilarity_local_ of each city was then partitioned into the components Nestedness_local_ and Turnover_local_. Lastly, we estimated the pairwise Jaccard dissimilarity for each urbanization level between two cities at a time, to test if distance affected dissimilarity. All calculations of dissimilarity, nestedness, and turnover were done using the R‐package *betapart* (Baselga et al., 2018).

### 2.6 Statistical analyses

We first assessed if there were any differences in habitat quality along the urbanization gradient, based on woodland size, Shannon's diversity index for trees, dead wood, and number of shrubs (see Nielsen et al., 2014). We also analyzed if the proportion of land covered by woodlands within the city limit (urban and suburban areas) was different from rural areas. Before analyzing the data, we averaged all the woodland features among woodlands within the same urbanization level in each city. For each woodland feature, as well as for the proportion of land covered by woodlands, we modelled the effect of urbanization level with linear mixed models fitted with restricted maximum likelihood with the R‐package *lme4* (Bates et al., 2015) after visually controlling that the model residuals were normally distributed and homoscedastic.

We assessed how bird diversity differed between urbanization levels at the regional scale by comparing the diversity metrics: γ_region,urban_; γ_region,suburban_; γ_region,rural_, α_region,urban_; α_region,suburban_; α_region,rural_, and β_region,urban_; β_region,suburban_; β_region,rural_. To assess differences in species composition between urbanization levels at the regional scale, we compared the Dissimilarity_region_, Nestedness_region_, and Turnover_region_, between urban and suburban areas, suburban and rural areas, and urban and rural areas. All calculations were carried out separately with the dataset containing all woodland‐breeding species and the subset with red‐listed species. These assessments did not involve any statistics because *n* = 1 for each metric.

We constructed species accumulation curves with the R‐package *vegan* (Oksanen et al., 2019) showing the effect of the sampled number of cities on the accumulating number of species, as well as red‐listed species. For this analysis, we aggregated the bird data across woodlands within the same urbanization level and city (*n* = 32 per urbanization level). We constructed separate species accumulation curves for urban, suburban, and rural areas to compare at how many cities the increase in species showed a pattern of flattening.

To assess differences in local bird diversity between urbanization levels, we analyzed the local γ‐, α‐, and β‐diversity metrics calculated for each individual city (*n* = 32) with linear mixed models using the R‐package *nlme* (Pinheiro et al., 2020) after visually controlling that the model residuals were normally distributed and homoscedastic. We included α _local,urban_; α _local,suburban_; α _local,rural_, and β _local,urban_; β _local,suburban_; β _local,rural_ in the same models (one model including all woodland‐breeding species and one the subset with red‐listed species) to analyze differences between urbanization levels and diversity components. Because of potential heteroscedasticity, we included individual variance estimates for urbanization level (urban, suburban, and rural) and diversity component (α and β), when these significantly improved the model fit: all species (LRT = 4.30, *p* = .231) and red‐listed species (LRT = 83.59, *p* < .001; see Zuur et al., 2009). We fitted separate models to assess the differences between γ_local,urban_, γ_local,suburban_ and γ_local,rural_ because of the inherent correlation between γ and the other diversity components (γ = α + β). As above, this was done in separate models for all woodland‐breeding species and the subset with red‐listed species. We verified that the variance did not differ significantly between γ_local,urban_, γ_local,suburban_ and γ_local,rural_ by comparing models with even and individual variance estimates for urbanization level: all species (LRT = 2.86, *p* = .239) and red‐listed species (LRT = 0.59, *p* = .745).

To assess differences in species composition between urbanization levels at the local scale, we analyzed Dissimilarity_local_ and its components Nestedness_local_ and Turnover_local_ between urban and suburban areas, suburban and rural areas, and urban and rural areas, of each city (*n* = 32). To this end, we used generalized linear mixed models. We fitted the models with beta‐distributed errors using a logit link function with the R‐package *betareg* (Zeileis et al., 2016). This required that we transformed the calculated Dissimilarity_local_, Nestedness_local_ and Turnover_local_ so that no values equaled 0 or 1, for which the beta distribution is not applicable. This was done by taking [values × (*n*–1) + 0.5]/*n*, where *n* denotes the total number of cities (i.e., 32; Smithson & Verkuilen, 2006). We included the transformed Nestedness_local_ and Turnover_local_ in the same models to analyze differences between compared urbanization levels and dissimilarity components. We fitted separate models with the transformed Dissimilarity_local_ because of the inherent correlation between the Jaccard dissimilarity and its components (dissimilarity = nestedness + turnover). We fitted separate models with diversity estimates for all woodland‐breeding and red‐listed species.

To assess if distance between cities affected dissimilarity, we modelled the impact of distance between city pairs (all possible pair combinations) on the dissimilarity for each of the urbanization levels. We used generalized linear mixed models fitted as above.

In all statistical models above, we included a random intercept term for city identity to account for the non‐independence among samples from the same city and different sampling efforts among cities. We used Tukey post hoc tests with the R‐package *emmeans* to retrieve *p*‐values (Lenth, 2020).

Ultimately, we analyzed the difference in probability of a species occurring in a woodland in urban and rural areas with risk ratios. Risk ratios express the ratio of the probability of a species occurring in an urban area compared with the probability of the same species occurring in a rural area. For instance, a risk ratio of 10 implies that a species is 10 times as likely to be found in a woodland in the urban area as in one in the rural area, while 0.1 implies the opposite. We calculated risk ratios with linear models with binomial error distributions using a log link function with the R‐package *lme4* (Bates et al., 2015). *p*‐values were retrieved with *z*‐tests. All statistical analyses and visualizations were performed with R version 4.0.0 (www.r‐project.org).

## 3 RESULTS

There were no significant differences between woodlands in urban, suburban, and rural areas regarding any of the woodland features measuring habitat quality: woodland size, Shannon's diversity index for trees, dead wood, and number of shrubs (*p* > .05; Tables S2 and S3). The proportion of land covered by woodland was higher in rural areas (44 ± 18% SD) compared with in urban and suburban areas (19 ± 9% SD; *t* = 9.91, *p* < .001; Table S3).

### 3.1 All species at the regional scale

We identified in total 59 woodland‐breeding bird species (Table S4), out of which all occurred in rural, 51 in suburban and 47 in urban areas (Figure S1). There were eight species unique to rural and no species unique to suburban or urban areas (Figure S1). Amongst the 47 species that occurred in both urban and rural areas, the probability to be observed in urban areas was higher for 7 species and lower for 18 species, compared with rural areas (Figure S2).

**FIGURE 2:**
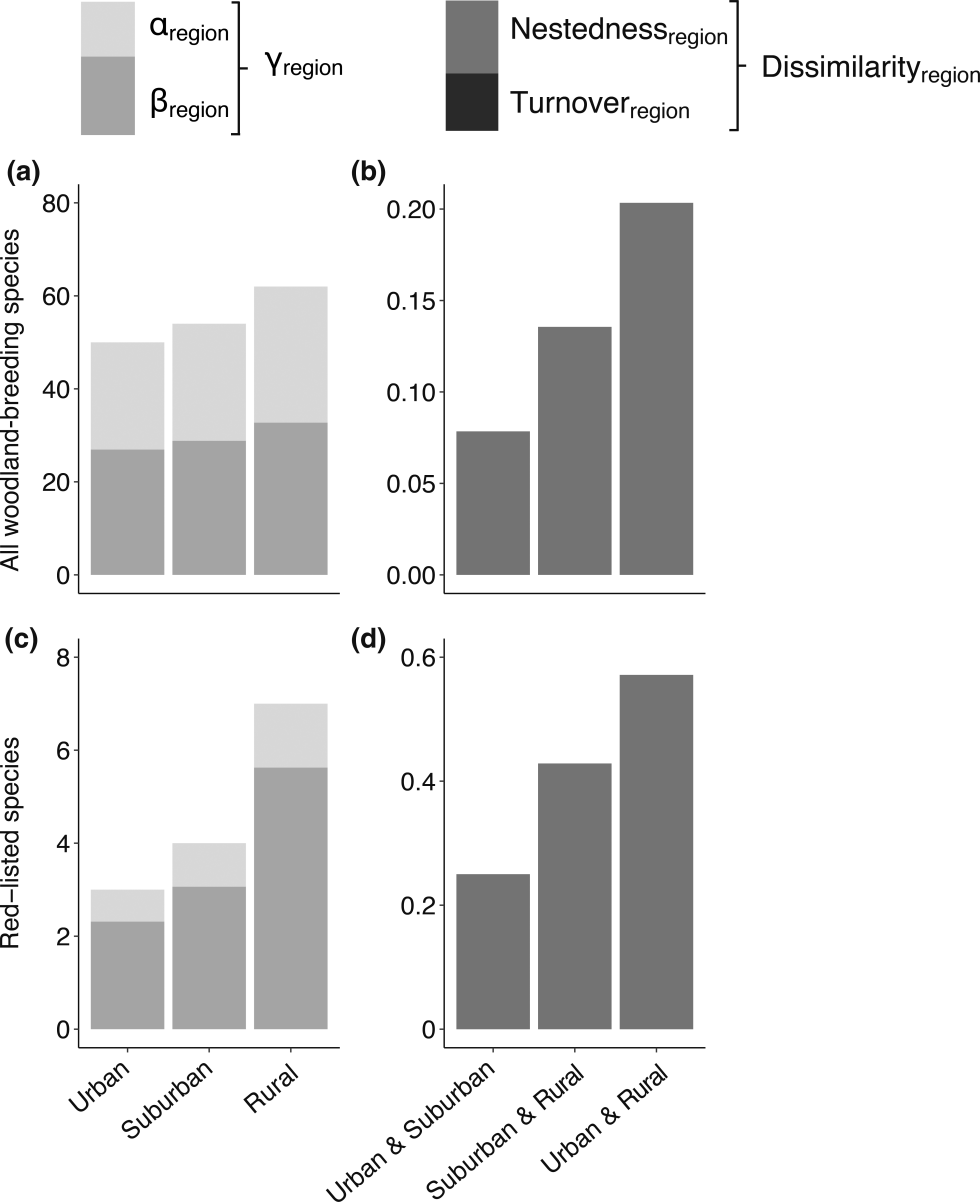
γ‐, α‐, and β‐diversity at the regional scale for all woodland‐breeding species (a) and the subset of red‐listed species (c) in different urbanization levels. Dissimilarity, nestedness, and turnover of all woodland‐breeding species (b) and red‐listed species (d) between different urbanization levels at the regional scale.

The total species richness (γ‐diversity) at the regional scale (across all cities), was highest in rural and lowest in urban areas (Figure 2a). This was to a lesser degree caused by differences in α‐diversity and to a larger degree by a decline in β‐diversity along the urbanization gradient (Figure 2a). Yet, β‐diversity was higher than α‐diversity for all three urbanization levels. Thus, the average number of species occurring in each urbanization level of a city comprised less than half of the total regional species pool in the same urbanization level.

The dissimilarity in region‐wide species composition was largest between urban and rural areas and smallest between urban and suburban areas (Figure 2b). The dissimilarity between urbanization levels was entirely due to a systematic loss of species (nestedness) along the urbanization gradient (Figure 2b). A greater distance between cities led to higher dissimilarity in species composition, but the impact of distance on dissimilarity did not differ between urbanization levels (Table S5; Figure S3).

### 3.2 Red‐listed species at the regional scale

Out of the 59 identified woodland‐breeding species, 7 were red listed at the time of the surveys according to the Swedish Red List (Table S4). Of these, all (three exclusively) occurred in rural, four (none exclusively) in suburban and three (none exclusively) in urban areas (Figure S1). Of the three species occurring in both rural and urban areas, two were most probable to be observed in a rural area, and for one the occurrence probability did not differ between urban and rural areas (Figure S2). We observed red‐listed species in the rural areas of 28 (88%) cities, in the suburban areas of 23 (72%) cities and in the urban area of 17 of the 32 (53%) cities.

For red‐listed species, γ‐diversity at the regional scale was largely caused by β‐diversity, which was considerably higher in rural than in suburban and urban areas (Figure 2c).

The dissimilarity in region‐wide species composition of red‐listed species was largest between urban and rural areas and smallest between urban and suburban areas (Figure 2d). All dissimilarity was caused by nestedness (Figure 2d). Dissimilarity between different urbanization levels was in all cases higher for red‐listed than all species.

### 3.3 Species accumulation

**FIGURE 3:**
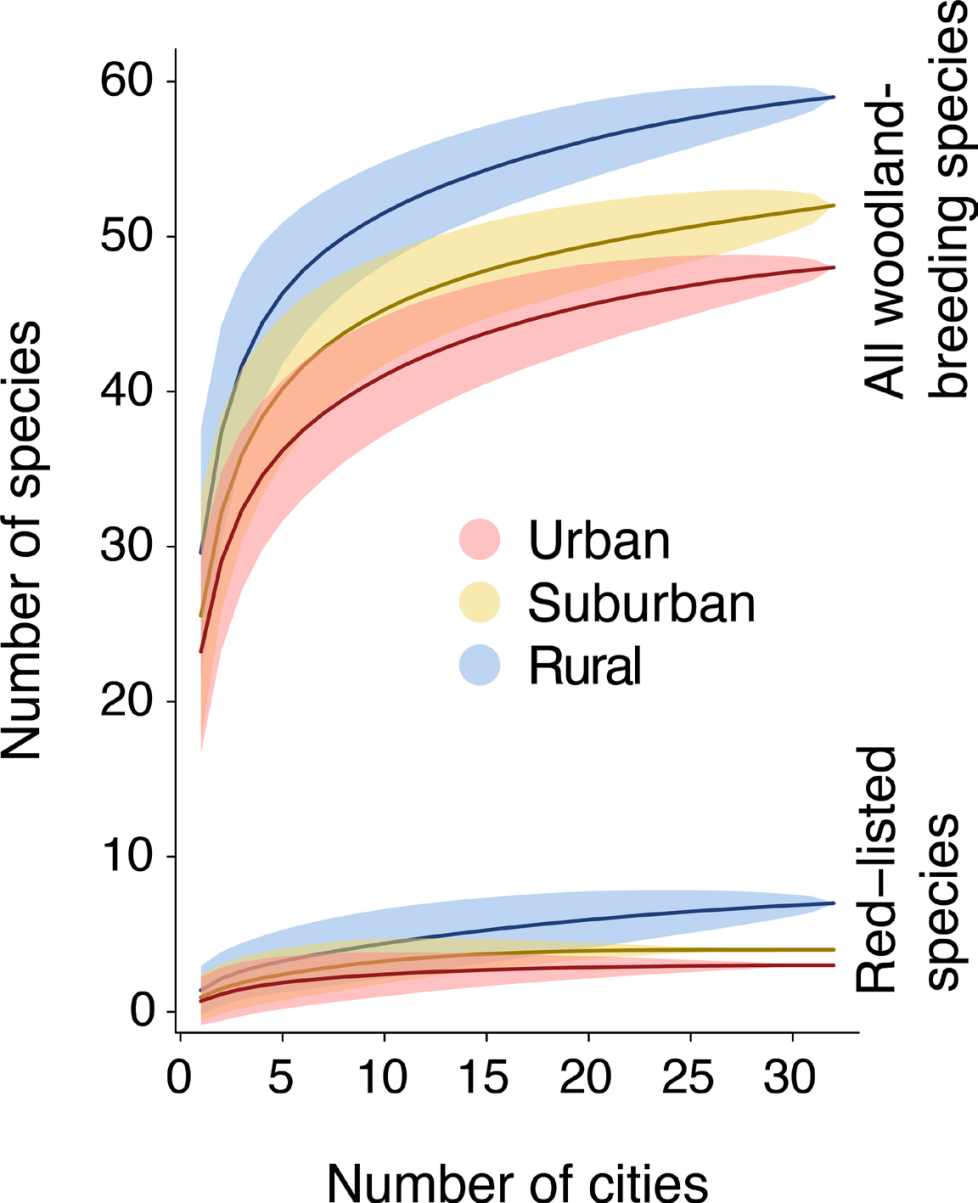
Species accumulation curves (mean estimate with 95% CI) for all woodland‐breeding species and red‐listed species in the different urbanization levels (*n* = 32).

The species accumulation curves show that the increase in cumulative species richness as more cities are studied is highest in rural and lowest in urban areas (Figure 3). The pattern is similar for red‐listed species; however, the relative difference between rural areas and the other two urbanization levels is greater (Figure 3). At the maximum number of cities (*n* = 32), a plateau stage is approached for the species accumulation curve of red‐listed species in urban and suburban areas (Figure 3). Thus, surveys in additional cities within the region could be expected to yield no additional red‐listed woodland‐breeding species in urban or suburban areas.

### 3.4 All species at the local scale

**FIGURE 4:**
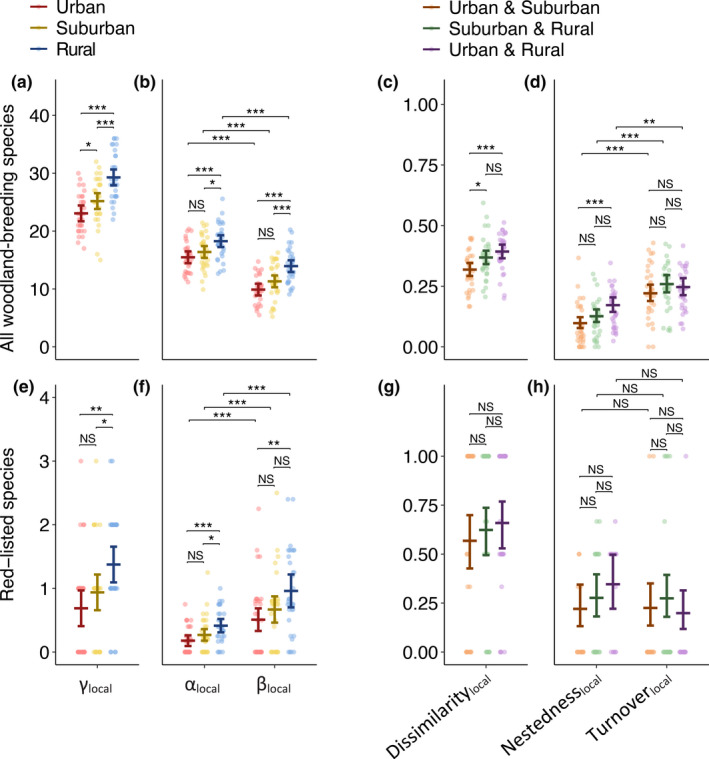
γ‐, β‐, and α‐diversity at the local scale for all woodland‐breeding species (a, b) and red‐listed species (e, f) in different urbanization levels. Dissimilarity, nestedness and turnover of all woodland‐breeding species (c, d) and red‐listed species (g, h) between different urbanization levels at the local scale. Error bars show estimated effects and 95% CI, points show raw data (*n* = 32). The estimated effects and CI are back transformed from log odds to proportions in c, d, g, and h. Statistical comparisons (see Tables S4 and S5) were done with linear mixed models and Tukey post‐hoc tests. NS, non‐significant (*p* > .05), *p < .05, **p < .01, ***p < .001.

The total species richness (γ‐diversity) at the local scale was significantly higher in rural areas than in both urban and suburban areas, and in suburban than in urban areas (Figure 4a; Table S6). The α‐ and β‐diversity were also significantly higher in rural areas than in the two more urbanized areas (Figure 4b; Table S6). In contrast to the regional scale, α‐diversity was higher than β‐diversity within each urbanization level (Figure 4b; Table S6). Thus, the average number of observed species per woodland comprised more than half of the total species community in the same urbanization level and city.

The dissimilarity in local species composition was significantly smaller between urban and suburban areas compared with the other pairwise comparisons (Figure 4c; Table S7). The nestedness between urban and suburban areas was significantly smaller than between urban and rural areas (Figure 4d; Table S7). While turnover did not differ between the pairwise compared urbanization levels, it was consistently larger than nestedness, indicating that the difference in species composition between urbanization levels within a city was to a larger extent explained by species replacement, rather than by a systematic species loss (Figure 4d).

### 3.5 Red‐listed species at the local scale

For red‐listed species, rural areas had a higher γ‐, α‐, and β‐diversity than urban areas, and γ‐ and α‐diversity than suburban areas (Figure 4e,f; Table S6). In contrast to when analyzing all woodland‐breeding species, β‐diversity of red‐listed species was significantly higher than α‐diversity at all urbanization levels (Figure 4f; Table S6). Thus, on average, the number of locally observed red‐listed species in a woodland represented less than half of the total species assemblage of red‐listed species observed in the same urbanization level and city. We found no differences in local scale dissimilarity, nestedness or turnover of red‐listed species between any of the pairwise compared urbanization levels (Figure 4g,h; Table S7).

## 4 DISCUSSION

Previous studies assessing effects of urbanization on bird diversity across spatial scales have included data from mixed habitats, such as woodlands, riparian areas, managed parks, and percentage intact vegetation (Aronson et al., 2014; Batáry et al., 2018; Ferenc et al., 2014). Compared with these studies, we kept the focal habitat constant (i.e., woodlands) and varied the urbanization level to disentangle the effects of urban landscapes from those of the surveyed habitats. Our results thus provide new insights into how urban landscapes affect bird communities irrespective of local habitat quality. We found that landscape urbanization decreases the diversity and heterogeneity of woodland bird communities, and that red‐listed species were particularly affected, no matter the spatial scale. Of particular interest for conservation, analyses at the regional scale suggested that dissimilarity between urbanization levels was caused by a systematic loss of species (nestedness) with increased urbanization, and at the local scale mainly by species turnover. Hence, our results show that urban and suburban woodlands contribute less to the regional species richness compared with rural woodlands, but that woodlands need to be retained in all urbanization levels to maximize local bird conservation.

### 4.1 Regional scale

Studies assessing the impact of urbanization on bird diversity at a regional scale (across multiple cities) have typically not differentiated between the impact of habitat quality and urban landscapes (e.g., Aronson et al., 2014; Ferenc et al., 2014). By using a unique dataset on bird diversity in woodlands with similar habitat quality independent on urban context (Hedblom & Söderström, 2010), we showed that urban landscapes per se are negatively associated with regional α‐, and (in particular) β‐diversity. Typically, β‐diversity relates positively to environmental heterogeneity (i.e., available niche space) among habitats (Stein et al., 2014). However, because the habitat quality of sampled woodlands was similar across the urbanization gradient, β‐diversity patterns were more likely driven by differences in environmental heterogeneity in the landscapes surrounding the woodlands (Santana et al., 2017). Rural areas around Swedish cities are to a varying degree dominated by forest, agricultural land, built‐up land, and wetlands (SCB, 2019), and the proportion of land covered by woodlands was in our dataset significantly higher in rural areas compared with urban and suburban areas. Because the landscape can affect species occurring in local habitats, for example, by providing supplementary and complementary resources (Smith et al., 2014), a larger variation and availability of resources in landscapes surrounding rural woodlands can increase the total niche space across cities, and thus the number of species occurring in woodlands. In comparison, land cover and other environmental conditions, such as climate and topography, are more homogenous across urban and suburban areas of different cities as they are organized and managed more similarly (Dronova, 2017; Groffman et al., 2014). In addition, the decline we found in average species richness per urbanization levels (regional α‐diversity) with increased urbanization, suggests that the urban and suburban landscapes of a given city provided less heterogenous and useful resources across woodland communities compared with rural areas. Thus, we argue that the capacity of urban landscapes surrounding woodlands to provide supplementary or complementary resources, most notably foraging habitats, constrains the diversity of birds breeding in urban woodlands.

In contrast to our results, some studies have found the bird diversity in suburban areas (or intermediate levels of urbanization) to be almost as high as in rural areas (Batáry et al., 2018), or even higher (Blair, 2004; Marzluff, 2008). However, because these studies did not control for habitat type, their results may be driven by a comparatively high habitat heterogeneity in suburban areas (McKinney, 2002), rather than the effect of landscape urbanization per se, which we have studied. In contrast, by controlling for habitat type and quality, we showed that an increase in landscape urbanization from rural to suburban had per se a negative impact on bird diversity. In fact, total species richness declined more between rural and suburban areas than between suburban and urban areas, irrespective of spatial scale. Furthermore, as nestedness was smallest between urban and suburban areas, our results indicate that species were mainly filtered out as urbanization increased from low levels.

Although several species were favored by urbanization (Figure S2, for similar results, see Taylor et al., 2016), we found no species strictly associated with urban or suburban areas. Instead, dissimilarities in species composition at the regional scale were entirely due to nestedness. This, as well as largely non‐overlapping confidence intervals in species accumulation curves between urban and rural areas, suggests a systematic loss of species, rather than species replacement, with increased urbanization (see also Persson et al., 2020). A systematic loss of species along an urbanization gradient is often mediated by environmental filtering against particular functional traits, such as insectivorous species (Callaghan et al., 2020; Marcacci et al., 2021). Indeed, many species that were unique to rural areas, or occurred in rural areas with a higher probability than urban areas, are insectivorous during breeding season when the surveys were conducted, for example, thrush nightingale (*Luscinia luscinia*), long‐tailed tit (*Aegithalos caudatus*), Eurasian treecreeper (*Certhia familiaris*), Eurasian wren (*Troglodytes troglodytes*), and dunnock (*Prunella modularis*). There were also several species known to have a low tolerance to humans during breeding season among the species unique to, or more probable to occur in, rural areas, for example, northern goshawk (*Accipiter gentilis*), common raven (*Corvus corax*), common cuckoo (*Cuculus canorus*), and Eurasian jay (*Garrulus glandarius*; BirdLife International, 2022). Because some of these species can forage over large areas, most notably the northern goshawk and the common raven, increasing disturbance associated with urbanization, such as noise and light pollution (Gilbert, 2012), may be the primary reason to why they were lacking in woodlands embedded in increasingly urban landscapes.

In addition, urbanization could potentially be particularly negative for species with a small distribution range, since this is often associated with a narrow niche breadth and high sensitivity to environmental change (Gaston, 1994). However, because distance between cities affected dissimilarity in species composition similarly for all urbanization levels, distribution range did not appear to affect species' sensitivity to urbanization. A contrasting result was found by Ferenc et al. (2014), where distance between sites was associated with a greater increase in dissimilarity in rural than in urban areas. Their result may rather be driven by concomitant greater habitat heterogeneity in rural than in urban areas (habitat quality was not controlled for), than a greater sensitivity to landscape‐scale urbanization among species distributed towards the extremes of the study area.

### 4.2 Local scale

Similar to the aggregated, regional scale, the higher β‐diversity in rural than urban and suburban areas at the local scale was likely explained by less heterogeneous landscapes and more disturbances within urban and suburban areas of a given city, compared with rural areas. The higher local α‐diversity in rural than urban and suburban areas is likely driven by a higher quality of complementary and supplementary resources in the landscape around the surveyed woodlands, possibly linked to the proportion of forest cover (Melles et al., 2003).

In opposite to the regional scale, the local α‐diversity of each urbanization level was consistently higher than the β‐diversity. These opposing relationships are most likely caused by relatively rare species disproportionally contributing to higher β‐diversity at larger spatial scales (Table S4, see also Socolar et al., 2016), and increasingly dissimilar species composition between sites further apart (Figure S3).

Also in contrast to the regional scale, the dissimilarity in species composition between any two compared urbanization levels at the local scale was to a larger extent caused by turnover than nestedness. Thus, differences in species composition along the urbanization gradient within a given city was mainly due to different occurring species rather than by a systematic loss in species from less to more urban areas.

Although the greater role of species turnover at the local than regional scale may in part be explained by sampling effects, caused by species occurring in only a few sites (see Table S4), it may also be explained by certain local or landscape resources that are often only found in one or two of a city's urbanization levels, while yet existing in all levels across cities. For instance, although local habitat features did not differ across the urbanization gradient in our study, the occurrence of some species may be tied to specific resources that only occur sporadically (e.g., cavities in high trees for nesting). In a similar vein, suitable open foraging habitats such as arable land or wetlands may only rarely occur in some urban or suburban contexts, which may limit many woodland birds from breeding in local woodland if such foraging habitats are lacking from the surrounding. As an example, the hawfinch (*Coccothraustes coccothraustes*) occurred in all urbanization levels (Table S4) and did therefore not affect the regional nestedness or turnover. Yet, it occurred in almost twice as many of the cities' urban than rural areas (increasing the local turnover), possibly because of more food resources, such as planted fruit trees.

### 4.3 Red‐listed species

To assess how urbanization affected species of conservation concern, we used the red‐list classification that was effective at the time of the surveys. Analyzes based on red‐listed species indicated that these were disproportionally affected by increasing urbanization; 43% of all red‐listed species existed exclusively in rural areas, and among those occurring in both urban and rural areas, 66% were more probable to occur in rural areas (Figures S1 and S2). Moreover, the relative declines in γ‐, α‐, and β‐diversity between rural and urban areas were larger for red‐listed species than all species at both the regional and local scale. The disproportional negative effect on red‐listed species may partly be caused by their in general smaller population sizes that make them less tolerable to habitat isolation (Pulliam, 1988) and environmental disturbances (Gaston, 1994), which may likely increase with increasing urbanization (Beninde et al., 2015; Snep et al., 2006). Furthermore, red‐listed species may to a larger extent possess traits that are negatively related to increasing urbanization (see Hedblom & Söderström, 2010). For instance, several invertebrate groups that are less abundant in urban areas (Piano et al., 2020) constitute important food resources to red‐listed species that only occurred in rural areas, or were more probable to occur in rural than urban areas, for example, European honey buzzard (*Pernis apivorus*), Eurasian golden oriole (*Oriolus oriolus*), and marsh tit (*Poecile palustris*; where the latter two are still red‐listed in Sweden).

It is notable that we found red‐listed species in a majority of cities' urban and suburban areas, in contrast to, for example, Aronson et al. (2014), where red‐listed species were only found in 14% of the sampled cities. The comparatively high occurrence of red‐listed species in our study may be explained by the fact that we controlled for local habitat quality as far as possible (cf., Aronson et al., 2014). Our results thus suggest that high‐quality habitats in urban and suburban areas can be important for maintaining local communities of red‐listed species, as long as they are not too intensively managed (Beninde et al., 2015). For instance, woodlands with mature deciduous trees is a principal habitat for the red‐listed species marsh tit and lesser spotted woodpecker (*Dryobates minor*), both of which occurred in a majority of cities' urban and suburban areas in our study. As marsh tit, lesser spotted woodpecker is still red listed in Sweden.

### 4.4 Conservation implications

An increasing rate of urban expansion in coming decades (Zhou et al., 2019) is expected to exacerbate the negative impact of urbanization on biodiversity (Seto et al., 2012). Natural and semi‐natural habitats play a critical role in maintaining biodiversity within and around cities, and understanding how they are affected by surrounding urbanization is thus of high importance for biodiversity conservation (Beninde et al., 2015; Melles et al., 2003).

Our study design allowed us to draw some important conclusions regarding the biodiversity impact of urban landscapes per se, independently of local habitat quality, as well as on the contribution of woodlands to the local and regional diversity depending on their location along an urbanization gradient. Firstly, to preserve the species richness of woodland‐breeding birds across a region encompassing multiple cities, we found that it is particularly important to maintain natural habitats in rural areas, where 14% of all bird species in our study exclusively occurred, including almost half of all red‐listed species. In addition, earlier research has suggested that woodlands and other (semi‐) natural habitats in rural areas can further play an important role in supporting immigration to suburban and urban habitats (Snep et al., 2006). Thus, our results show that urban expansion may negatively affect the regional bird diversity if natural habitats are exploited, as well as if rural areas surrounding natural habitats are urbanized.

Secondly, based on our local‐scale analyses, woodlands in all urbanization levels are important to maintain a high local species richness, as implied by (i) the fact that 7 and 18 species were more likely to be observed in urban or rural woodlands, respectively, and (ii) the significantly higher species turnover than nestedness explaining the species dissimilarity across the urbanization gradient (see also Dale, 2018). The large role of species turnover at the local scale suggests that unique sites are important for local bird diversity, and much more so than what appears to be the case based on regional‐scale analyses. This is further supported by the local β‐diversity indicating a high importance of individual woodlands for the species richness of each urbanization level in a city. This was particularly true for red‐listed species, where β‐diversity was significantly higher than the α‐diversity in each urbanization level. Thus, to maintain a high species pool in cities, it is therefore important to preserve existing woodlands along the entire urbanization gradient, in particular sites containing unique species. Such sites may be surrounded by landscapes that can offer substantial complementary or supplementary resources, such as water bodies or various open habitats that are not too intensively managed.

Because the data were collected in 2004, we note that the quality of the surveyed woodlands, and the urbanization level of their surrounding landscapes, may have changed with ensuing consequences for the bird communities. Based on satellite images from 2021, approximately 7% of the woodlands have been exploited to some extent since the surveys were conducted (7 urban, 13 suburban, and 10 rural woodlands), with possible consequences for these woodlands' bird communities. Thus, the contrasts between urbanization levels may have changed over time, for example, a greater loss of sensitive species in suburban woodlands because of the higher exploitation rate, but the exact effect will also depend on to which extent the surrounding landscapes have changed. Although we believe that our results have general conservation implications, similar studies in different contexts are needed to verify their generality. To facilitate biodiversity consideration in urban planning, there is a need for further assessments on the impact of landscape urbanization depending on habitat type and quality and, based on our findings, on how to reduce negative impacts of increasing urbanization on biodiversity in (semi‐) natural habitats (Dronova, 2017).

## 5 CONCLUSION

In summary, we demonstrated that urbanization reduced bird species richness and homogenized species communities in woodlands with constant habitat quality and that red‐listed species were particularly affected. Our results suggest that the negative effects which urbanization had on bird communities were mainly driven by a decreased capacity of the surrounding landscape to provide complementary and supplementary resources, possibly in combination with increasing human disturbance filtering out certain species. Important for conservation, we showed that the dissimilarities in species composition between urbanization levels were at the regional scale caused by a systematic loss of species in more urban landscapes, and at the local scale largely by species replacement. Hence, we conclude that woodland habitats in rural areas are the most important for the regional‐wide species richness, but that habitats in all urbanization levels are needed to maximize the local bird diversity.

## CONFLICT OF INTEREST

The authors have no conflict of interest to declare.

## Supporting information


Appendix S1

## Data Availability

The data that support the findings of this study are openly available in dryad at https://doi.org/10.5061/dryad.08kprr54f.
