# Supplementary material for: Urbanization causes biotic homogenization of woodland bird communities at multiple spatial scales
Source: Glob Chang Biol. 2022 Aug 14;28(21):6152–64. doi: 10.1111/gcb.16350 (PMC9804485; doi:10.1111/gcb.16350)
Supplement: Supplementary file 1 — Appendix S1 [file GCB-28-6152-s001.pdf]

Supporting information to:
Urbanization causes biotic homogenization of woodland bird communities at multiple spatial scales

TABLE S1. Surveyed cities

City	Human pop. Size (2004)	Size of city (ha)	Woodlands per urbanization level
Alingsås	23000	1081	4
Borås	63000	3131	6
Enköping	38000	1031	4
Eslöv	17000	870	4
Falköping	16000	801	4
Gislaved	10000	706	4
Göteborg	510000	19908	6
Hässleholm	18000	1202	5
Karlshamn	19000	1525	4
Köping	17000	1220	5
Kumla	13000	744	4
Kungsbacka	18000	853	6
Landskrona	29000	1126	3
Lerum	16000	1490	5
Lidingö	30000	1314	5
Ljungby	15000	1012	5
Malmö	258000	6645	5
Mölnlycke	13000	770	4
Örebro	98000	4383	5
Skara	11000	649	4
Skövde	33000	1996	5
Södertälje	60000	2500	5
Stockholm	1252000	40005	6
Täby	59000	2649	5
Trollhättan	44000	2506	5
Tumba	35000	1835	5
Uppsala	128000	4905	6
Vallentuna	27000	1484	4
Vänersborg	21000	1242	4
Värnamo	18000	1116	5
Västerås	107000	5007	6
Växjö	56000	2895	5

TABLE S2. Descriptive statistics of habitat quality of woodlands

Predictor	Mean	Standard deviation
Shannon's diversity for trees	0.57	0.43
dead wood quantity	5.38	9.76
shrub quantity	10.41	15.41

TABLE S3. The estimated difference in habitat quality of woodlands and forest cover between different urbanization levels. The relationships were analyzed with general mixed effect models fitted with restricted maximum likelihood. P values were retrieved with Tukey post hoc tests.

Response variable	Urbanization level	Estimate	Standard error	t value	P value
woodland size	urban vs. suburban	42.08	21.64	1.94	0.135
	urban vs. rural	4.55	21.64	0.21	0.976
	suburban vs. rural	46.63	21.64	2.15	0.087
Shannon's diversity for trees	urban vs. suburban	0.05	0.05	0.97	0.599
	urban vs. rural	0.02	0.05	0.41	0.912
	suburban vs. rural	0.03	0.05	0.56	0.843
dead wood quantity	urban vs. suburban	0.22	0.90	0.25	0.966
	urban vs. rural	1.28	0.90	1.42	0.337
	suburban vs. rural	1.06	0.90	1.17	0.475
shrub quantity	urban vs. suburban	1.31	2.03	0.64	0.797
	urban vs. rural	2.28	2.03	1.12	0.504
	suburban vs. rural	0.97	2.03	0.48	0.881
forest cover	urban and suburban vs. rural	0.25	0.03	9.91	**<0.001**

TABLE S4. Woodland-breeding bird species and their red-list status (if threatened or near threatened) and the number of cities in which they occurred in the urban, suburban and rural area respectively. The table also shows the number of woodlands in the urban, suburban and rural area that bird species occurred in.

Species			Number of cities and (woodlands) with occurrences		
English	Latin	Red-list status	Urban	Suburban	Rural
Black woodpecker	*Dryocopus martius*		2 (2)	3 (3)	15 (17)
Brambling	*Fringilla montifringilla*		6 (8)	8 (9)	7 (10)
Coal tit	*Periparus ater*		5 (5)	11 (15)	12 (20)
Common blackbird	*Turdus merula*		32 (141)	32 (134)	32 (138)
Common buzzard	*Buteo buteo*		0 (0)	0 (0)	12 (17)
Common chaffinch	*Fringilla coelebs*		32 (152)	32 (149)	32 (153)
Common chiffchaff	*Phylloscopus collybita*		5 (9)	8 (14)	8 (15)
Common cuckoo	*Cuculus canorus*		1 (1)	7 (7)	15 (22)
Common raven	*Corvus corax*		2 (2)	1 (1)	13 (17)
Common redpoll	*Acanthis flammea*		1 (1)	4 (4)	2 (2)
Common redstart	*Phoenicurus phoenicurus*		18 (36)	15 (24)	9 (14)
Common starling	*Sturnus vulgaris*		25 (61)	27 (66)	27 (61)
Common wood pigeon	*Columba palumbus*		29 (121)	32 (115)	32 (127)
Dunnock	*Prunella modularis*		13 (19)	15 (27)	24 (44)
Eurasian blackcap	*Sylvia atricapilla*		31 (82)	29 (85)	30 (105)
Eurasian blue tit	*Cyanistes caeruleus*		32 (145)	32 (143)	32 (125)
Eurasian bullfinch	*Pyrrhula pyrrhula*		2 (3)	5 (6)	4 (6)
Eurasian eagle-owl	*Bubo bubo*	NT	0 (0)	0 (0)	1 (1)
Eurasian golden oriole	*Oriolus oriolus*	EN	0 (0)	0 (0)	2 (2)
Eurasian jay	*Garrulus glandarius*		12 (14)	16 (25)	20 (34)
Eurasian nuthatch	*Sitta europaea*		31 (116)	30 (114)	32 (107)
Eurasian siskin	*Spinus spinus*		19 (33)	20 (38)	21 (43)
Eurasian sparrowhawk	*Accipiter nisus*		2 (2)	3 (3)	3 (4)
Eurasian treecreeper	*Certhia familiaris*		10 (16)	17 (22)	22 (43)
Eurasian woodcock	*Scolopax rusticola*		0 (0)	1 (1)	1 (1)
Eurasian wren	*Troglodytes troglodytes*		13 (19)	14 (32)	19 (38)
Eurasian wryneck	*Jynx torquilla*	NT	0 (0)	3 (3)	2 (2)
European crested tit	*Lophophanes cristatus*		3 (6)	4 (5)	9 (13)
European goldfinch	*Carduelis carduelis*		5 (9)	8 (12)	15 (22)
European green woodpecker	*Picus viridis*		16 (22)	24 (38)	26 (53)
European greenfinch	*Chloris chloris*		32 (137)	32 (128)	32 (122)
European honey buzzard	*Pernis apivorus*	EN	0 (0)	0 (0)	1 (1)
European pied flycatcher	*Ficedula hypoleuca*		31 (103)	31 (97)	24 (65)
European robin	*Erithacus rubecula*		31 (112)	31 (126)	32 (124)
Fieldfare	*Turdus pilaris*		32 (134)	32 (120)	32 (89)
Garden warbler	*Sylvia borin*		23 (36)	24 (60)	28 (64)
Goldcrest	*Regulus regulus*		22 (43)	22 (47)	29 (77)
Great spotted woodpecker	*Dendrocopos major*		22 (41)	24 (50)	28 (69)
Great tit	*Parus major*		32 (152)	32 (150)	32 (146)
Green sandpiper	*Tringa ochropus*		0 (0)	1 (1)	5 (5)
Hawfinch	*Coccothraustes coccothraustes*		20 (51)	17 (35)	12 (22)
Icterine warbler	*Hippolais icterina*		8 (10)	8 (10)	7 (13)
Lesser spotted woodpecker	*Dryobates minor*	VU	5 (5)	4 (4)	3 (3)
Long-tailed tit	*Aegithalos caudatus*		0 (0)	0 (0)	1 (1)
Marsh tit	*Poecile palustris*	NT	15 (19)	19 (30)	21 (34)

Mistle thrush	*Turdus viscivorus*		3 (4)	1 (1)	7 (8)
Northern goshawk	*Accipiter gentilis*		0 (0)	0 (0)	2 (3)
Parrot crossbill	*Loxia pytyopsittacus*		0 (0)	0 (0)	2 (4)
Red crossbill	*Loxia curvirostra*		1 (1)	1 (1)	1 (2)
Red kite	*Milvus milvus*		0 (0)	0 (0)	2 (3)
Redwing	*Turdus iliacus*		20 (29)	18 (34)	21 (41)
Song thrush	*Turdus philomelos*		28 (58)	29 (70)	32 (108)
Spotted flycatcher	*Muscicapa striata*		12 (14)	9 (12)	15 (23)
Stock dove	*Columba oenas*	VU	2 (3)	4 (4)	14 (19)
Tawny owl	*Strix aluco*		1 (1)	1 (1)	2 (2)
Thrush nightingale	*Luscinia luscinia*		2 (2)	8 (15)	14 (30)
Willow tit	*Poecile montanus*		0 (0)	2 (2)	8 (10)
Willow warbler	*Phylloscopus trochilus*		31 (133)	32 (135)	32 (143)
Wood warbler	*Phylloscopus sibilatrix*		18 (31)	23 (43)	19 (31)

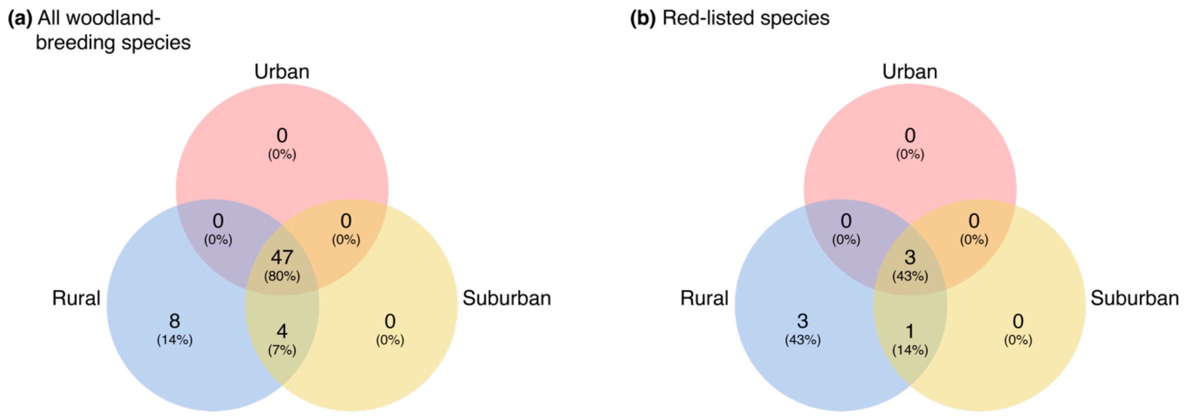

FIGURE S1. Venn diagrams showing the number and percentage of all woodland-breeding species (a) and red-listed species (b) that occurred in several and unique urbanization levels.

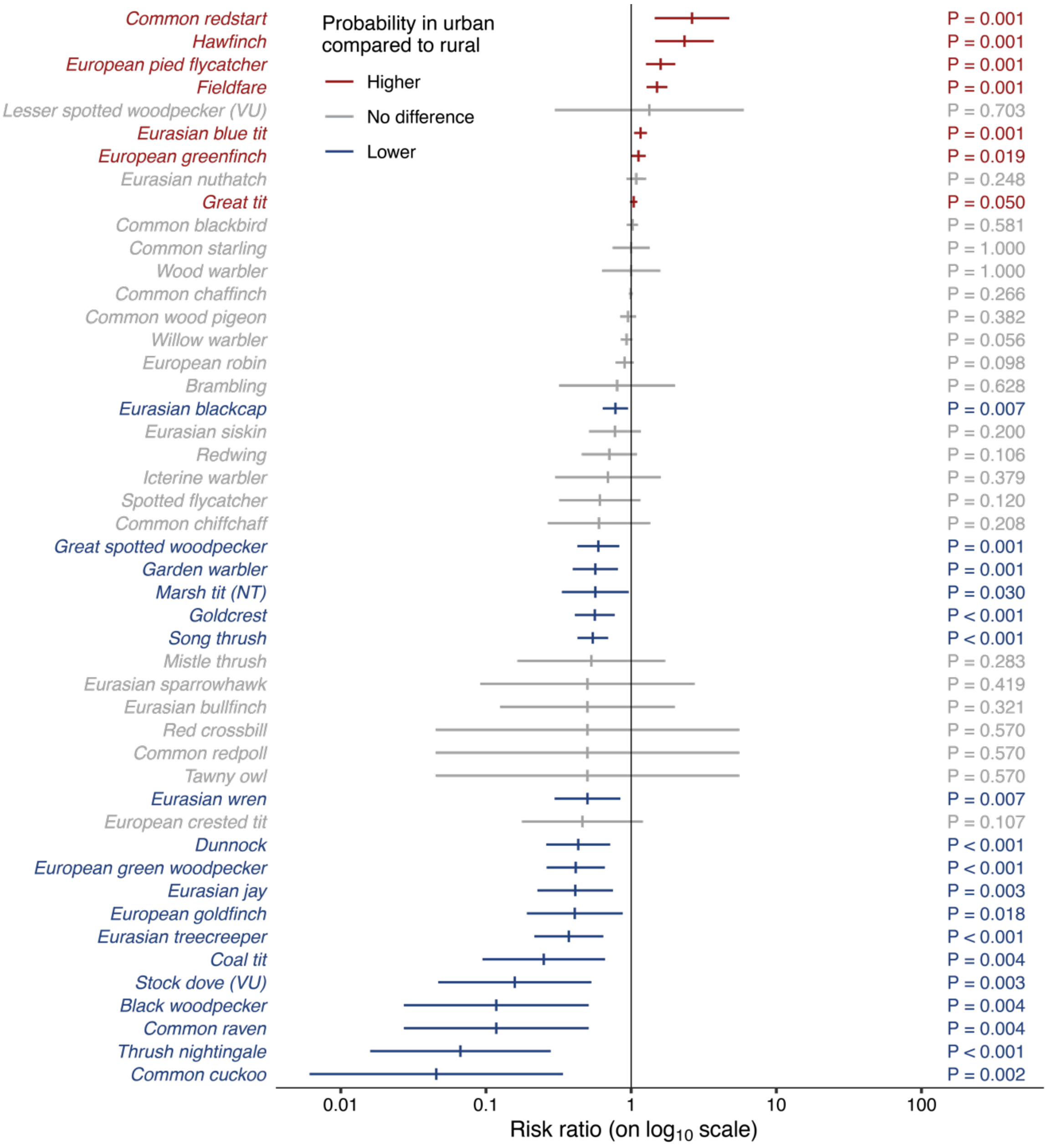

FIGURE S2. Risk ratio of woodland-breeding species (red-list status of threatened and near threatened species in parenthesis) that were observed in both urban and rural areas. The error bars show the estimated probability and 95% CI of that a species is observed in urban compared to rural areas. Species that are typed in red are significantly more probable to be observed in urban than rural areas, grey are as probable to be observed in urban as in rural areas and blue are significantly less probable to be observed in urban than rural areas.

TABLE S5. The effect of distance (km) between cities on the dissimilarity in woodland-breeding species composition, and the difference in dissimilarity between different urbanization levels, as well as the interaction effect of distance and urbanization level on dissimilarity. The interaction effect investigates if the impact of distance on dissimilarity is dependent on urbanization level and is not represented by a regression coefficient. The relationships were analyzed with generalized mixed effect models fitted with restricted maximum likelihood. P values were retrieved with Tukey post hoc tests on the logit scale.

Predictor	Estimate	Standard error	t value	P value
Jaccard dissimilarity				
Distance	0.0007	0.00008	9.26	**<0.001**
urban vs. suburban	0.05	0.02	2.44	**0.015**
urban vs. rural	0.06	0.02	3.43	**<0.001**
suburban vs. rural	0.02	0.02	0.99	0.321
distance × urbanization level			1.24	0.539

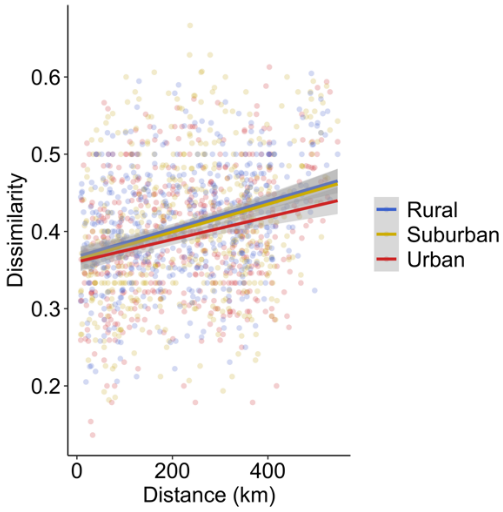

FIGURE S3. The relationship between distance between city pairs and dissimilarity in woodland-breeding species composition in urban, suburban and rural areas. Lines and shaded areas show estimated effects and 95% CI, points show raw data (n = 496 per urbanization level). The estimated effects and CI are back transformed from log odds to proportions. Statistical comparisons (see Table S5) were done with linear mixed models and Tukey post hoc tests.

TABLE S6. The estimated difference in gamma, alpha and beta diversity between different urbanization levels, and the difference between alpha and beta diversity in urban, suburban and rural areas respectively. All woodland-breeding species and red-listed species were modelled separately. The relationships were analyzed with general mixed effect models fitted with restricted maximum likelihood. P values were retrieved with Tukey post hoc tests.

Contrast					
**Diversity component**	**Urbanization level**	**Estimate**	**Standard error**	**t value**	**P value**
Model with gamma for all woodland-breeding species					
gamma	urban vs. suburban	2.13	0.70	3.02	**0.010**
	urban vs. rural	6.22	0.70	8.84	**<0.001**
	suburban vs. rural	4.09	0.70	5.82	**<0.001**
Model with alpha and beta for all woodland-breeding species					
alpha	urban vs. suburban	0.83	0.60	1.46	0.760
	urban vs. rural	2.53	0.60	4.45	**<0.001**
	suburban vs. rural	1.70	0.60	3.99	**0.029**
beta	urban vs. suburban	1.29	0.60	2.27	0.199
	urban vs. rural	3.69	0.60	6.47	**<0.001**
	suburban vs. rural	2.39	0.60	4.20	**0.001**
alpha vs. beta	urban	5.01	0.60	8.92	**<0.001**
	suburban	4.61	0.60	8.10	**<0.001**
	rural	3.92	0.60	6.89	**<0.001**
Model with gamma for red-listed species					
gamma	urban vs. suburban	0.25	0.18	1.37	0.361
	urban vs. rural	0.69	0.18	3.77	**0.001**
	suburban vs. rural	0.44	0.18	2.40	**0.050**
Model with alpha and beta for red-listed species					
alpha	urban vs. suburban	0.09	0.04	2.13	0.088
	urban vs. rural	0.24	0.05	4.74	**<0.001**
	suburban vs. rural	0.15	0.05	2.76	**0.018**
Beta	urban vs. suburban	0.16	0.13	1.26	0.419
	urban vs. rural	0.45	0.15	3.06	**0.007**
	suburban vs. rural	0.29	0.16	1.87	0.151
alpha vs. beta	urban	0.33	0.09	3.84	**<0.001**
	suburban	0.40	0.10	3.92	**<0.001**
	rural	0.55	0.13	4.21	**<0.001**

TABLE S7. The estimated difference in nestedness, turnover and dissimilarity between different urbanization levels, and the difference between nestedness and turnover in urban, suburban and rural areas respectively. All woodland-breeding species and red-listed species were modelled separately. The relationships were analyzed with generalized mixed effect models fitted with restricted maximum likelihood. P values were retrieved with Tukey post hoc tests.

Contrast					
**Diversity component**	**Urbanization level**	**Estimate**	**Standard error**	**t value**	**P value**
Model with Jaccard dissimilarity for all woodland-breeding species					
Jaccard	urban & suburban vs. suburban & rural	0.22	0.08	2.73	**0.020**
	suburban & rural vs. urban & rural	0.33	0.08	4.04	**<0.001**
	urban & suburban vs. urban & rural	0.10	0.08	1.31	0.391
Model with nestedness and turnover for all woodland-breeding species					
nestedness	urban & suburban vs. suburban & rural	0.29	0.17	1.68	0.216
	suburban & rural vs. urban & rural	0.65	0.16	2.30	0.059
	urban & suburban vs. urban & rural	0.36	0.17	3.94	**<0.001**
turnover	urban & suburban vs. suburban & rural	0.21	0.14	1.53	0.280
	suburban & rural vs. urban & rural	0.14	0.14	1.05	0.548
	urban & suburban vs. urban & rural	0.06	0.13	0.48	0.879
nestedness vs. turnover	urban & suburban	0.96	0.16	5.99	**<0.001**
	suburban & rural	0.88	0.15	5.87	**<0.001**
	urban & rural	0.46	0.14	3.19	**0.002**
Model with Jaccard dissimilarity for red-listed species					
Jaccard	urban & suburban vs. suburban & rural	0.23	0.33	0.69	0.770
	suburban & rural vs. urban & rural	0.38	0.34	1.15	0.487
	urban & suburban vs. urban & rural	0.15	0.32	0.49	0.876
Model with nestedness and turnover for red-listed species					
nestedness	urban & suburban vs. suburban & rural	0.30	0.40	0.75	0.732
	suburban & rural vs. urban & rural	0.63	0.43	1.45	0.320
	urban & suburban vs. urban & rural	0.33	0.41	0.80	0.705
turnover	urban & suburban vs. suburban & rural	0.26	0.40	0.66	0.789
	suburban & rural vs. urban & rural	0.16	0.42	0.38	0.925
	urban & suburban vs. urban & rural	0.42	0.40	1.06	0.541
nestedness vs. turnover	urban & suburban	0.03	0.42	0.06	0.950
	suburban & rural	0.01	0.37	0.03	0.974
	urban & rural	0.76	0.43	1.76	0.082
